# HERV-K10 as a mediator of immune modulation in hepatitis infections

**DOI:** 10.3389/fimmu.2025.1624774

**Published:** 2025-09-17

**Authors:** Salih Özer, Romano Strobelt, Anna D. Kosinska, Goar Frishman, Jochen M. Wettengel, Lisa Pleninger, Nina Körber, Wen Liang, Edanur Ates Öz, Marisol Zuniga, Tanja Bauer, Gregor Ebert, Ulrike Protzer, Michelle Vincendeau

**Affiliations:** ^1^ Institute of Virology, Helmholtz Munich, Munich, Germany; ^2^ Institute of Virology, School of Medicine and Health, Technical University Munich, Munich, Germany; ^3^ Institute of Experimental Genetics, Helmholtz Munich, Munich, Germany; ^4^ German Center for Infection Research (DZIF), Munich, Germany

**Keywords:** HERVs, hepatitis, Gag1/MAG1, HERV-K10, immune modulation, autoimmune diseases

## Abstract

The human genome contains ~8% of endogenous retroviruses (HERVs), whose reactivation has been implicated in diseases such as cancer and autoimmune disorders. Among these, HERV-K10 has attracted attention for its potential role in immune modulation and viral infections. This study investigates HERV-K10 expression in hepatitis virus infections, focusing on its impact on host gene expression and immune responses. We analyzed HERV-K10 in PBMCs from patients chronically infected with hepatitis C virus (HCV) and in HBV-infected liver cell models. Our results show a significant upregulation of HERV-K10 in HBV-infected HepG2-NTCP cells, HCV-infected PBMCs, and a trend in HBV-infected primary hepatocytes. HERV-K10 activation was specific to hepatitis infection, as no effect was seen with HBV entry inhibitors, adenovirus 5 infection or infection with other RNA viruses. RNA sequencing of HBV-infected HepG2-NTCP cells revealed distinct clustering based on HERV expression profiles, including HERV-K10 encoding the MAG1 domain, an immune response target. To investigate the potential immunomodulatory role of HERV-K10 MAG1, we vaccinated mice with the MAG1 peptide, which resulted in activation of CD4+ and CD8+ T-cell responses and higher levels of MAG1-specific antibodies. Furthermore, chronic hepatitis B patients exhibited an immune response to MAG1 characterized by elevated levels of Interleukin-6 (IL-6) and interleukin-1β (IL-1β) cytokines. Taken together, our data suggest that HERV-K10 plays an important role in immune modulation during viral hepatitis infection and may contribute to the pathogenesis of autoimmune diseases.

## Introduction

Autoimmune diseases are a complex and diverse group of disorders in which the body’s immune system mistakenly attacks its own tissues. These conditions can affect various organs, including the skin. A hallmark of autoimmune diseases is the production of autoantibodies that target self-antigens, leading to inflammation and tissue damage (1). While the precise mechanisms underlying these diseases are not fully understood, factors such as environmental triggers and immune system dysregulation are known to play a significant role in their development (2). Autoimmune diseases are often associated with chronic infections, such as those caused by hepatitis B virus (HBV) or hepatitis C virus (HCV) (3). Chronic infection with these viruses can disrupt the function of the immune system, triggering the production of autoantibodies and immune complexes that mistakenly attack the body’s own tissues. Hepatitis virus infections have been associated with several autoimmune diseases, including mixed cryoglobulinemia (MC), lupus-like symptoms, and rheumatoid arthritis (RA) (3). Molecular mimicry is thought to play a role in the development of these diseases (4). This hypothesis suggests that viral proteins may share structural similarities with host proteins and that antibodies against viral antigens may cross-react with physiological ones, leading the immune system to mistakenly target the body’s own tissues, resulting in autoimmune joint damage (5). Besides exogenous viruses, human endogenous retroviruses (HERVs) have also been implicated in driving molecular mimicry and autoimmune diseases (6–11). Bioinformatic analyses have shown sequence similarity between IgG1-Fc and specific peptide sequences of the group-specific antigen 1 (gag1) of HERV-K10 (11). RA patients showed significant levels of cross-reactive antibodies against both gag1 and IgG-Fc, suggesting that the autoimmune response may be mediated by molecular mimicry. Typically, HERVs are silenced by epigenetic mechanisms (6, 12, 13). However, HERVs can be reactivated by environmental triggers such as viral infections (14–17). This reactivation may contribute to autoimmune responses through molecular mimicry, adding another layer of complexity to the pathogenesis of autoimmune disease following infection (14, 17, 18). In this study, we aimed to investigate the molecular mechanisms linking hepatitis infection, HERVs, and immune responses. A deeper understanding of how viral infection, immune dysregulation, and molecular mimicry contribute to autoimmunity may pave the way for novel therapeutic strategies that address the underlying causes of these diseases, ultimately offering hope for improved management and treatment options for patients with autoimmune diseases.

## Materials and methods

### Tissue culture

HepG2-NTCP-K7 cells were cultivated as already described (19). Briefly, cells were kept on Collagen R 0.2% coated (Serva, Heidelberg, Germany, Cat. No. 47254.01) T75 flasks in DMEM, high glucose (Life Technologies, Paisley, UK, Cat. No. 11960-044) containing 10 U/mL Penstrep (Life Technologies, NY, USA, Cat. No. 15140-122), 1 µM Sodiumpyruvate (Life Technologies, Paisley, UK, Cat. No. 11360-039), MEM NEAA (Life Technologies, Paisley, UK, Cat. No. 11140-035), 2 mM L-Glutamine (Life Technologies, Paisley, UK, Cat. No. 25030-024) and 10% FCS (Life Technologies, Paisley, UK, Cat. No. A5256701). HepaRG-NTCP-K7 cells were cultivated in Williams E (Life Technologies, NY, USA, Cat. No. 12551-032) containing 10% Hyclone FCS (Life Technologies, Paisley, UK, Cat. No. SH30066.03), 10 U/mL Penstrep, 2 mM L-Glutamine, 0.07 mg/mL Gentamicin (Ratiopharm, Ulm, Germany, Cat. No. PZN-03928180), 0.0225 I.E./mL human Insulin (Sanofi, Paris, France, Cat. No. PZN-05961106) and 0.0912 mg/mL Hydrocortisone (Pfizer Pharma, Berlin, Germany, Cat. No. PZN-01877030) as already described (20). Huh7 cells were cultivated in DMEM with high glucose and HEPES (Life Technologies, Paisley, UK, Cat. No. 21063-029) supplemented with 10% FCS (Life Technologies, Paisley, UK, Cat. No. A5256701), 10 U/mL penstrep (Life Technologies, NY, USA, Cat. No. 15140-122) and 1 µM sodiumpyruvate (Life Technologies, Paisley, UK, Cat. No. 11360-039).

### HBV infection

For HBV infection of HepG2-NTCP-K7 cells, cells were seeded at a density of 1.16 x 10^5^ cells/cm^2^, differentiated for 48 hours in cultivation media containing 2.5% DMSO (Sigma-Aldrich, St. Louis, USA, Cat. No. 34869). After differentiation cells were infected with a MOI of 200 vp/cell with purified HBV particles (21). The infection inoculate contains 4% PEG 6000 (Sigma-Aldrich, Darmstadt, Germany, Cat. No. 8.07491.1000) and 2.5% DMSO. It was kept on cells for indicated time points, washed with PBS and subsequent analysis was performed (RNA and DNA extraction, supernatant for HBeAg ELISA). For HBV infection of HepaRG-NTCP cells, cells were seeded at a density of 1.05 x 10^5^ cells/cm^2^ and differentiated for two weeks in cultivation media containing 1.8% DMSO. After differentiation, cells were infected with HBV-wt as HepG2-NTCP-K7 with the difference in infection inoculate of 1.8% DMSO and 5% PEG 6000. The duration of inoculation was 16 hours and cells were washed with PBS afterwards.

### RNA-virus infection

The hepatic cell line Huh7 was seeded at a density of 0.1 x10^6^ cells/well within 24-well plates. Next morning, cells were infected in triplicate with severe acute respiratory coronavirus 2 (SARS-CoV-2; EPI_ISL_406862) or tick-borne encephalitis virus hypr-strain (TBEV) with a multiplicity of infection (MOI) of 0.1. The inoculum were left for two hours and replaced with fresh growth medium after PBS wash step.

### Quantitative real-time PCR

Total RNA was isolated using the RNeasy Kit (Quiagen, Hilden, Germany, Catalogue Number 74104) followed by DNase I treatment (Thermo Scientific, Vilnius, Lithuania, Cat. No. EN0521). 1 µg of DNase treated total RNA was used for reverse transcription with the RevertAid RT Kit (Fisher Scientific, Schwerte, Germany, Cat. No. K1691). QRT-PCR was performed using Light Cycler 480 SYBR Green Master I (Roche Diagnostics, Mannheim, Germany, Cat. No. 04887352001) as described previously (22). Relative quantification was calculated utilizing RNA Polymerase II (RPII) as a housekeeping gene (22). Following Primer Sequences were used (5’-3’): RPII: FW: GCACCACGTCCAATGACA, REV: GTCGGCTGCTTCCATAA; HERV-K10: FW: TTGCCCATGGTTTCCAGAAC, REV: AGCTGCTTTAATAATGGCCC; pregenomicRNA (pgRNA): FW: TGTTCAAGCCTCCAAGCT, REV: GGAAAGAAGTCAGAAGGCAA; SARS-CoV-2: FW: GCCTCTTCTCGTTCCTCATCAC, REV: AGCAGCATCACCGCCATTG, TBEV: FW: GGGCGGTTCTTGTTCTCC, REV: ACACATCACCTCCTTGTCAGACT.

### HAdV-C5 generation

The generation of the HAdV-C5-reporter virus was performed as described previously (21). The same generation was used for the generation of HAdV-C5-MAG1 (21). Final virus stocks were concentrated through repeated centrifugation at 1000 rcf using the Amicon^®^ filter system (50 kDA, Millipore^®^). For determining the concentration of the virus stock, TCID_50_ (50% tissue culture infectious dose) was calculated by adding 5-fold serial dilution to HEK293A cells. Three days later, cells were fixed with 4% PFA, washed with PBS, and stained with 1% crystal violet solution to visualize the confluency of cells and to determine the concentration of infectious units (IU/mL).

### Animal experiments

Male C57BL/6 mice, 8 weeks old and with the haplotype H-2b/b (Janvier Labs), were maintained under pathogen-free conditions. Mice were intramuscularly injected with two doses of 2x10^9^ IU/ml AdV-C5 (in 100 µl PBS) four weeks apart. For murine sera collection, blood was taken through the v. facialis, collected through Microvette^®^ 500 Lithium-Heparin-Gel capillary blood collection tubes (Sarstedt^®^) and centrifuged at 10000 x g for 5 min. The well-being and weight of the mice were monitored weekly throughout the whole duration of the experiment.

### Isolation of spleen lymphocytes

Murine spleens were isolated and passed through a 100 µm nylon cell strainer using RPMI medium (Gibco^®^). Afterwards, tubes were centrifugated at 1500 rpm at 4°C for 5 min. Red blood cells were lysed by resuspending the cell pellet in 2 ml Ammonium-Chloride-Potassium buffer (150 mM NH_4_Cl, 10 mM KHCO_3_, 0.1 mM Na_2_EDTA in H_2_O, pH 7.3). The reaction was stopped with 45 ml RPMI medium after 1 minute and samples were centrifuged at 1500 rpm at 4°C for 5 min. Next, cells were resuspended in RPMI growth medium (10% FBS, 1% P/S; Gibco^®^) and filtered through a 100 µm cell strainer.

### Isolation of liver lymphocytes

Mouse liver was perfused with PBS before harvesting and isolating the cells through a 100 µm cell strainer using RPMI medium (Gibco^®^). After centrifugation at 1500 rpm at 4°C for 5 min, cells were resuspended in 8 ml 1 mg/ml collagenase type IV (Worthington^®^) RPMI solution and incubated at 37°C for 30 min. Next, 37 ml ice-cold RPMI medium was added and centrifugated at 1500 rpm at 4°C for 5 min. The pellet was resuspended in 3 ml 40% Percoll-PBS solution (GE Healthcare^®^) and transferred gently to new tubes containing 3 ml 80% Percoll-PBS solution (GE Healthcare^®^). To separate cells from debris and erythrocytes, the samples were centrifuged at 2600 rpm with the brake disengaged and the middle layer enriched in lymphocytes was transferred to a new tube with 40 ml RPMI medium. Finally, after two centrifugation-wash cycles, cells were mixed with 200 µl RPMI growth medium containing 1% P/S (Gibco^®^) and 10% FBS (Gibco^®^).

### Intracellular cytokine staining

Liver or spleen lymphocytes were incubated with MAG1 (RIGKELKQAGRKGNI, Peptides&Elephants^®^), Ad5V DNA-binding protein (FALSNAEDL, Peptides&Elephants^®^), or non-immunogenic ovabulmin A (OVA, SIINFEKL Peptides&Elephants^®^) together with 1 µg/ml brefeldin A (BFA, Sigma-Aldrich^®^) for 14h. Next, cells were stained for surface markers CD4 (eBioscience^®^), and CD8 (BD Bioscience^®^) using fluorophore conjugated antibodies and a Fixable Viability Dye eF780 (eBioscience^®^) to exclude dead cells from the analysis. Afterwards, cells were fixed and permeabilized using Cytofix/Cytoperm Kit (BD Bioscience^®^). After staining intracellular cytokines with fluorophore-conjugated antibodies against Tumor Necrosis Factor alpha (TNF) (Biolegend^®^) and Interferon gamma (IFNγ) (eBioscience^®^), samples were analyzed with CytoFlexS flow cytometer (Beckman Coulter^®^) and FlowJo software (Tree Star^®^).

### Enzyme-linked immunosorbent assay

A 96-well Nunc MaxiSorp plate was coated with either 200 ng of MAG1 peptide (RIGKELKQAGRKGNI, Peptides&Elephants^®^) or 5x10^5^ heat-inactivated Ad5V particles diluted in 100 µl/well carbonated buffer (15 mM Na_2_CO_3_ and 35 mM NaHCO_3_ in H_2_O, pH 9.6). Next morning, plates were washed with PBS with 0.05% Tween (PBST) and blocked with PBS containing 5% FBS for one hour. After another washing step, murine sera were diluted in 1:100 in PBS and added to coated wells for 2h. After washing with PBST the secondary goat anti-mouse-IgG antibody conjugated with horseradish peroxidase (Sigma-Aldrich^®^) diluted in 100 µl PBS with 1% FBS was added. After 1 hour incubation plates were washed with PBST after 1h and 100 µl TMB substrate (Invitrogen™) was added, incubated in the dark and stopped with 100 µl 2N sulfuric acid for optical density measurement at 450 nm following background substraction at 560 nm.

### Isolation and cryopreservation of peripheral blood mononuclear cells

Blood from healthy control subjects (n=5) and chronic hepatitis B patients was drawn with the Vacutainer CPT™ System into sodium citrate CPT tubes (Becton Dickinson Biosciences, USA), and tubes were mixed five times before storing them upright at room temperature. Within two hours of blood collection, CPT tubes were centrifuged in a horizontal rotor (swing-out head) (1800g, 15 min, RT). Next, plasma was removed, and PBMC were transferred to 15 mL polystyrene Falcon tubes and mixed with 10 ml PBS by gently inverting the tubes five times. As previously described (23), PBMC were centrifuged (300 g, 10 min, RT) twice in 10 ml of PBS. For counting, cells were resuspended in CTL Test medium (CTL Europe GmbH, Germany) and 10 μl of the cell suspension was diluted 1:2 with CTL Live/Dead cell counting dye (CTL-LDC Live/Dead cell counting kit, CTL Europe, Germany). Ten microliters of the stained cell suspension were pipetted into the counting chamber and cell counting was performed on an ImmunoSpot Ultimate UV Image analyzer (CTL Europe, Germany). Next, 5 × 10^6^ PBMC were cryopreserved per vial in 1.8 mL cryotubes (Thermo Scientific, Denmark) at a concentration of 1 × 10^7^ PBMC per 1mL freezing medium (fetal calf serum (FCS) (Life Technologies, Germany), supplemented with 10% DMSO (Sigma-Aldrich, Germany), using a freezing container (Thermo Scientific, Denmark) and stored at −80°C. After 24 h, PBMC were stored in the vapor phase of a liquid nitrogen tank until further use.

### MAG1 PBMC stimulation and cytokine multiplex assay

One million PBMCs from chronic HBV infected patients or healthy individuals were resuspended in 200 µl of growth medium with or without 0.5 µg/ml MAG1 peptide (RIGKELKQAGRKGNI, Peptides&Elephants^®^). After 22 h, the supernatant was analyzed for secreted cytokines using LEGENDplex^™^ human anti-virus response panel (Biolegend^®^) following manufacturer protocol and analysis software.

### HERV annotation, RNAseq processing, and differential expression analysis

HERV annotation, RNAseq processing, and differential expression analysis were performed as previously described in (14, 17). Briefly, repetitive sequences in the human genome (hg38) were identified using RepeatMasker and downloaded from the UCSC Table Browser (24). LTRs and transposon internal portions were annotated based on Kojima’s transposon nomenclature (25), and transposable element annotations were obtained from the HERVd database (26). Reads were aligned to the Ensembl human reference genome (GRCh38) using the STAR aligner (27), allowing up to 20 alignments and two mismatches per read. BAM files were processed with SQUIRE to quantify HERV expression at the locus level. The output was integrated with HERV annotation to generate read counts. Differential expression analysis was performed using DEseq2 (28), combining HERV and gene count metrics.

### Gene function and network analysis

REACTOME pathways (29) and Disease enrichment analysis for identified gene sets (**Supplementary Table 1**) upon HBV infection was conducted using the Set Analyzer tool, provided by the Comparative Toxicogenomics Database (CTD) and based on the CTD MEDIC disease library (30). A manually curated gene-disease/phenotype interaction network for autoimmune diseases was obtained from the CIDeR database (31).

### Statistical analysis

Statistics were generated using the GraphPad Prism software. Error bars represent standard deviation, *t*-tests are always two-tailed, and Tukey *post-hoc* test was applied for all ANOVA analyses. If not specified otherwise, the single values within graphs represent independent biological replicates. Schemes were partially done with NIAID Visual & Medical Arts (03/10/2025), in particular: NIAID BIOART Source for spleen (https://bioart.niaid.nih.gov/bioart/243), liver (https://bioart.niaid.nih.gov/bioart/230), and cryo blood vial (https://bioart.niaid.nih.gov/bioart/87).

### Ethics statement

Studies on material of human origin were approved by the ethics committee of the Technical University of Munich (approval number 548/15 S), and informed consent was obtained from all patients/participants. Mice experiments followed the European Health Law of the Federation of Laboratory Animal Science Associations, the German regulations of the Society for Laboratory Animal Science, and the 3R rules and were approved by the local Animal Care and Use Committee of Upper Bavaria (permission number: ROB-55.2-2532.Vet_02-24-7).

## Results

### Hepatitis infection increases expression of HERV-K10

**Figure 1:**
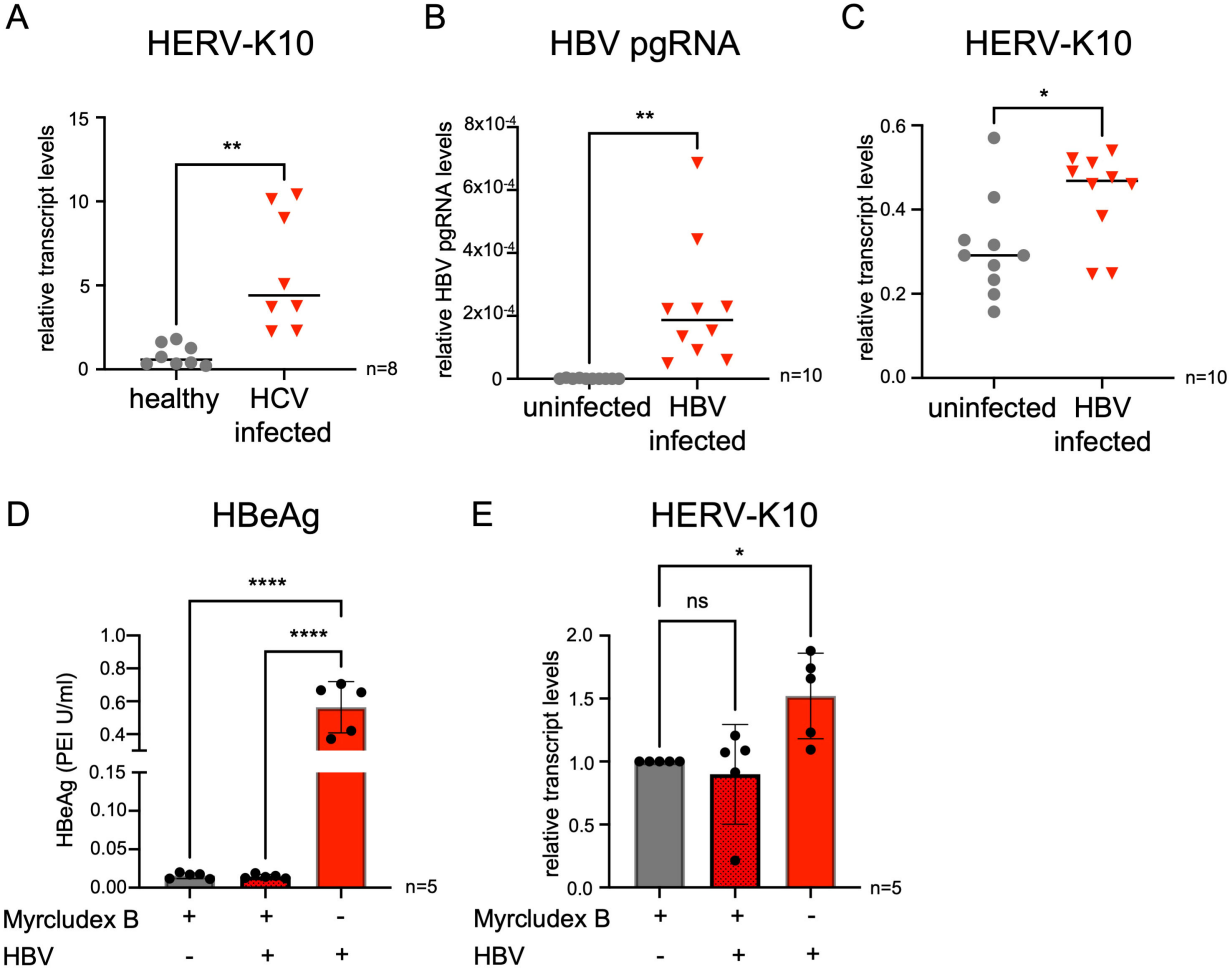
HERV-K10 mRNA levels are upregulated in PBMCs from chronic HCV patients and in HBV infected HepG2-NTCP-K7 cells. **(A)** Relative HERV-K10 mRNA expression in PBMCs from chronically HCV infected patients measured via qRT-PCR. **(B)** Relative pgRNA expression levels of HBV infected HepG2-NTCP-K7 on day two post infection. **(C)** Relative HERV-K10 mRNA expression of HBV infected HepG2-NTCP-K7 on day two post infection. **(D)** HBeAg levels in uninfected and HBV-infected HepG2-NTCP-K7 cells treated and untreated with Myrcludex **(B, E)** Relative HERV-K10 mRNA expression of HBV infected HepG2-NTCP-K7 with and without Myrcludex B treatment. For statistical analysis two-way-ANOVA was used (*p ≤ 0.05; **p ≤ 0.01); Data are mean ± SD ****p ≤ 0.0001 to the two-way ANOVA.

A range of autoimmune phenomena has been linked to hepatitis virus infections, yet the precise mechanisms through which these infections trigger autoimmune diseases remain poorly understood. To explore whether activation of HERV-K10, which has already been associated with autoimmune diseases (10, 11), might contribute to immune responses during hepatitis infection, we assessed HERV-K10 expression in primary blood mononuclear cells (PBMCs) infected with hepatitis C virus (HCV). Our analysis revealed that HERV-K10 transcript levels were significantly upregulated in PBMCs from patients with chronic HCV infection (16), compared to control samples from healthy individuals (**Figure 1A**). To further investigate the role of HERV-K10 in the context of hepatitis infection, we established an *in-vitro* model of hepatitis B virus (HBV) infection using the HBV-susceptible HepG2-NTCP-K7 cell line (19). Following successful HBV infection (**Figure 1B**), we observed a significant upregulation of HERV-K10 transcript levels two days post HBV- infection (**Figure 1C**). Additionally, in primary hepatocytes (PHH) from three different donors infected with HBV (**Supplementary Figure S1A**), we noted a trend towards increased HERV-K10 expression between three- and 10-hours post-infection (**Supplementary Figure S1B**).

To determine whether the upregulation of HERV-K10 is specifically triggered by HBV infection, HepG2-NTCP-K7 cells were pretreated with Myrcludex B, a clinically approved HBV entry inhibitor (32). Blocking viral entry effectively prevented HBV infection (**Figure 1D**) and abolished HERV-K10 upregulation (**Figure 1E**), whereas cells exposed to HBV without entry inhibition showed elevated HERV-K10 expression. These findings indicate that active viral entry is required for the induction of HERV-K10. To further evaluate viral specificity, we infected HepG2-NTCP-K7 cells with adenovirus 5 (Ad5V), a DNA virus. Despite successful infection, we observed no significant increase in HERV-K10 transcript levels (**Supplementary Figures S1C, D**). Next, we examined other RNA viruses, including SARS-CoV-2 and tick-borne encephalitis virus (TBEV), which strongly activate innate immunity. Although the cells were efficiently infected by the respective viruses (**Supplementary Figure S1E**), none showed a significant upregulation of HERV-K10 expression (**Supplementary Figure S1F**). These results suggest that HERV-K10 upregulation is not a general consequence of viral infection or innate immune stimulation, but rather a response specific to hepatitis viruses. Overall, our findings suggest that HERV-K10 activation is specifically associated with HBV and HCV infection.

### Transcriptome analysis in HBV-infected cells reveals upregulation of HERV-K10 and immune genes

**Figure 2:**
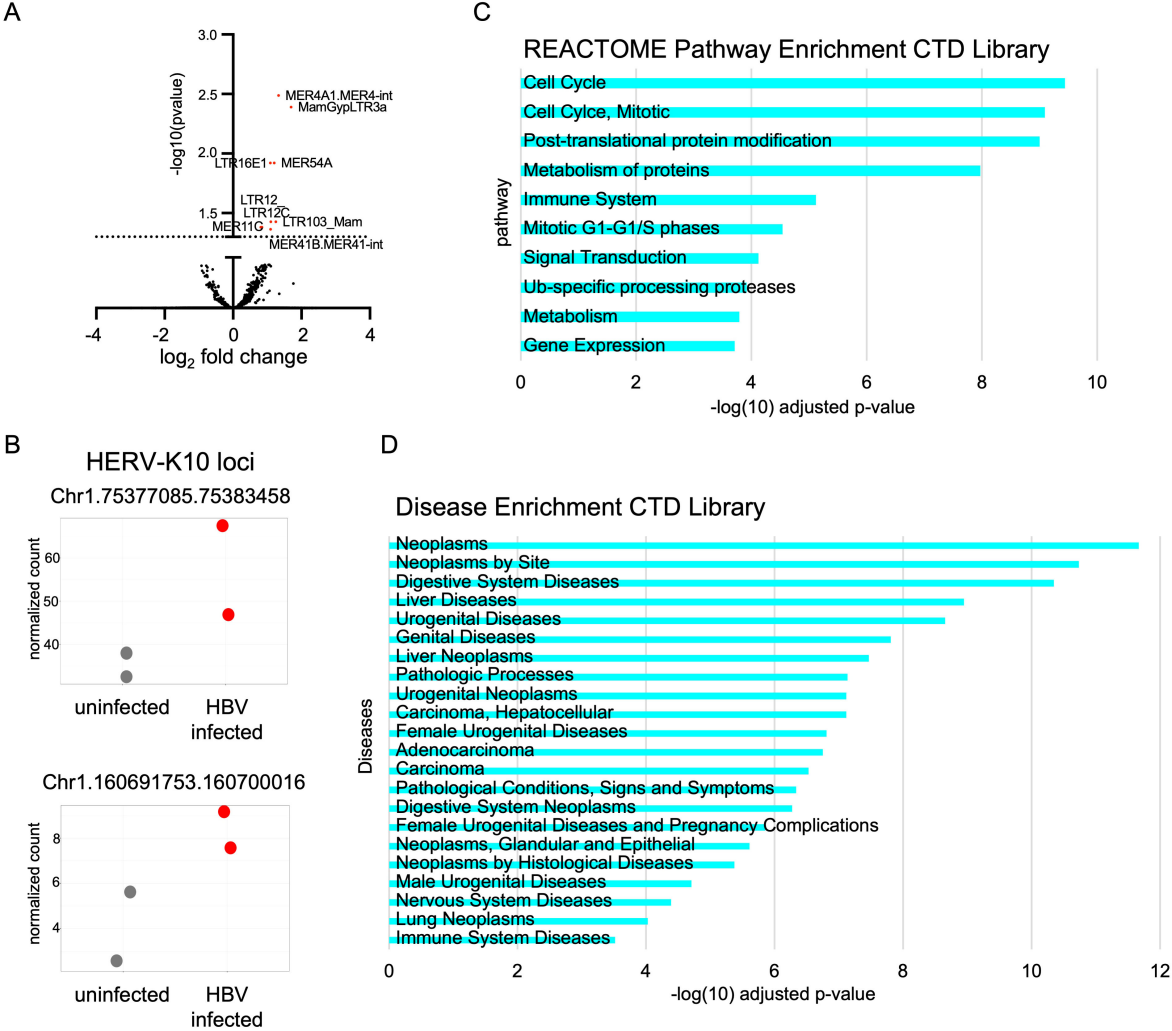
Various HERV groups and immune-related genes are activated upon HBV infection. **(A)** Principal component analysis (PCA) of HBV-infected HepG2-NTCP-K7 compared to non-infected control on day two post infection. The volcano plot shows differentially expressed HERV groups. **(B)** Shown are normalized read counts of the MAG1-containing HERV-K10 loci on chromosome 1 (160691753–160700016 and 75377085 – 75383458) of HBV infected versus non-infected control HepG2-NTCP-K7. Each data point represents an independent biological replicate. **(C)** REACTOME pathway enrichment analysis for differentially expressed genes induced by HBV infection. **(D)** Disease enrichment analysis for differentially expressed induced by HBV infection. **(C, D)** The bar graphs show the top categories from the CTD MEDIC disease library, all with adjusted p-values < 0.05.

To explore the interplay between HBV infection and HERV expression, we conducted a transcriptome analysis of HBV-infected HepG2-NTCP-K7 cells. Two days post-infection, distinct clustering of HERV expression profiles was observed in HBV-infected cells compared to non-infected controls (data not shown). Differential expression analysis revealed significant upregulation of several HERV groups, including LTR12 and MER41 (**Figure 2A**), both of which have previously been implicated in virus-induced transcriptional changes and regulation of immune-related genes (33). Although the LTR5 group—encompassing HERV-K10 loci—did not reach statistical significance at the group level, locus-specific analysis identified two individual HERV-K10 loci on chromosome 1 that were significantly upregulated upon HBV infection (**Figure 2B**). Interestingly, both HERV-K10 loci, encode the matrix and gag1 peptide (MAG1) domain—a known target of adaptive immune responses (9–11). In addition to the differentially expressed HERVs, we also identified 237 differentially expressed genes (both up- and down-regulated) in response to HBV infection (**Supplementary Figure S2A**, **Supplementary Table 1**). Detailed analysis of these genes revealed their involvement in key cellular processes, including cell cycle, immune regulation, metabolism, and signal transduction (**Figure 2C**). Notably, several genes were enriched in pathways associated with immune regulation and signal transduction, which are likely to be critical in the host’s attempt to control HBV infection while coping with viral-induced changes in gene expression, but could also trigger immune-related pathways relevant to autoimmune diseases. Disease enrichment analysis of the differentially expressed genes revealed their significant involvement in several diseases, including several forms of cancer, but also diseases of the immune system (**Figure 2D**). Several identified genes are associated with autoimmune diseases, including rheumatoid arthritis and systemic lupus (**Supplementary Figure S2B**). To further investigate potential regulatory interactions between HERVs and innate immune genes, we focused on genes located in the vicinity of HERV-K10 LTR5Hs elements. This analysis identified three candidate genes - TRIM56, JAGN1, and PRLR - that are known to play a role in immune response and viral restriction (34–36). Overall, we identified a clear clustering of differentially expressed HERV loci upon HBV infection, including LTR12C, LTR5, MER41, and HERV-K10. In addition, 237 genes were differentially expressed, many of which are involved in immune regulation, inflammation, and are associated with various autoimmune diseases.

### Induction of MAG1-specific immune responses in vaccinated mice and PBMCs of chronic hepatitis B patients

**Figure 3:**
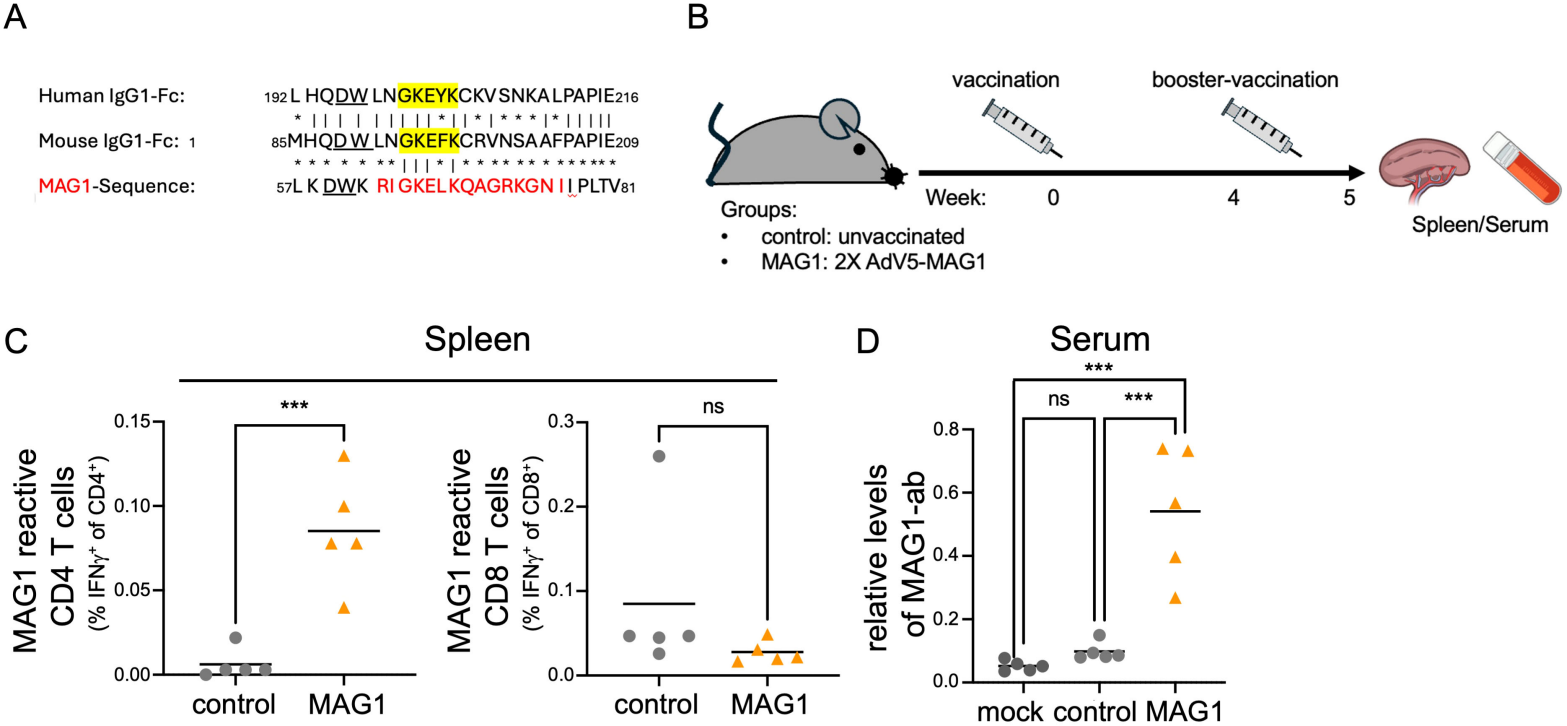
HERV-K10 derived MAG1-vaccination triggers CD4-specific immune response in mice. **(A)** Alignment of HERV-K10 MAG1 domain and human and murine IgG-Fc. MAG1-domain is depicted in red. GKEFK-peptide is marked in yellow. **(B)** Scheme of HAdV-C5-MAG1 vaccine regime. **(C)** Spleen lymphocytes were stimulated with MAG1 peptide for 14h. Afterwards, cells were stained for T-cell subclasses, intracellular IFNγ, and analyzed via flow cytometry. **(D)** The presence of MAG1-specific antibodies was tested in mouse sera using ELISA. For statistical analysis two-way-ANOVA was used (***p ≤ 0.001).

**Figure 4:**
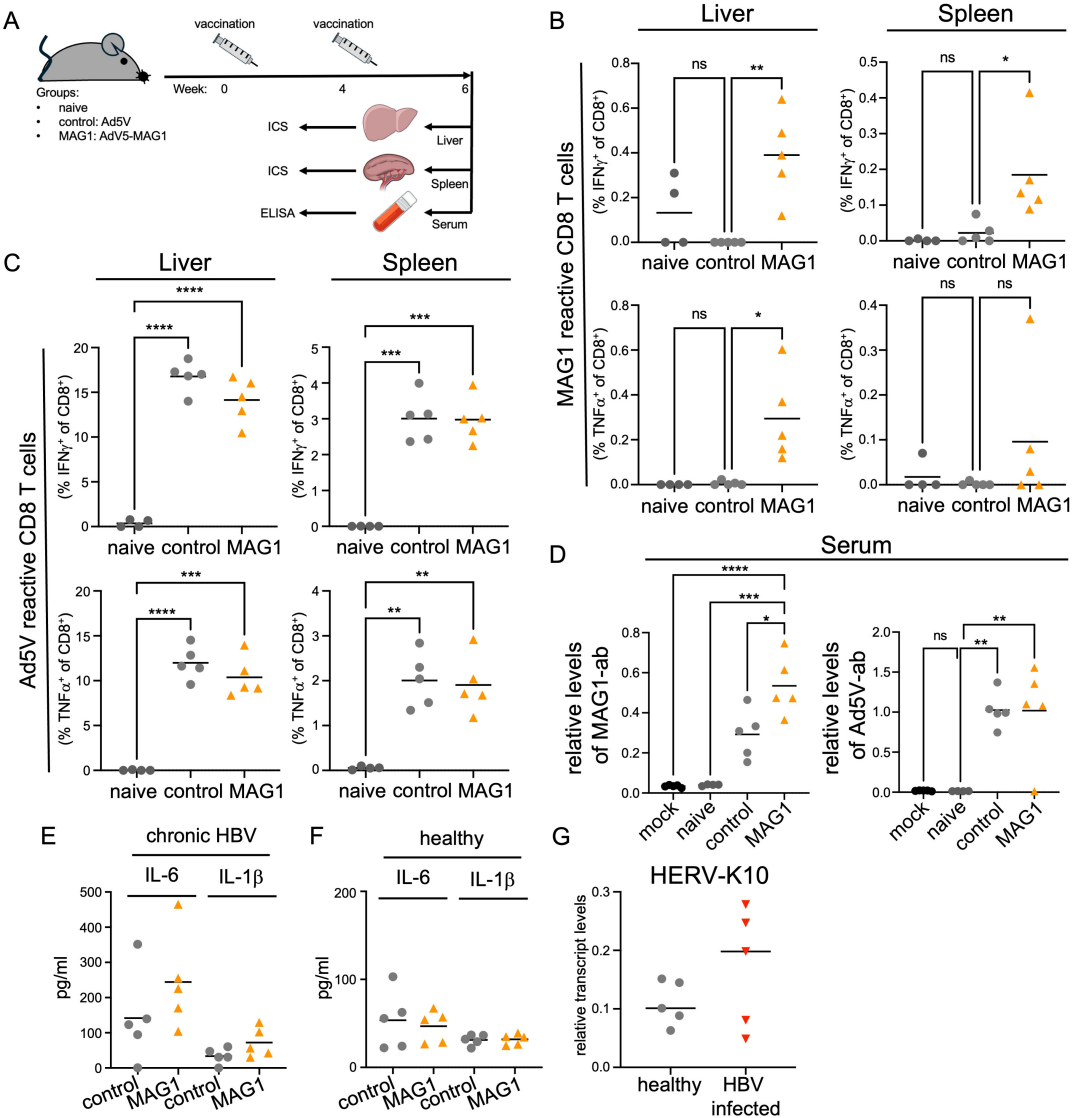
MAG1-specific immune response can be detected in mice and chronic HBV patients. **(A)** Scheme of HBV infection, vaccination regime, and analyses. Two weeks after the second vaccination, all mice were sacrificed to harvest liver, spleen, and serum samples for further analysis. **(B)** Liver or spleen lymphocytes from MAG1 vaccinated mice were stimulated with MAG1 peptide or control and stained for CD8 and intracellular IFNγ or TNFα via flow cytometry. **(C)** Murine spleen or liver lymphocytes were stimulated with Ad5V-dbp peptide, stained, and analyzed using flow cytometry. **(D)** MAG-1 peptide-specific antibodies were detected in mouse sera via ELISA. **(E, F)** Interleukin-6 (IL-6) and interleukin-1β (IL-1β) levels were measured in PBMCs of chronically HBV-infected patients **(E)** or healthy control PBMCs **(F)** after stimulation with a MAG-1 peptide or mock control using a cytokine multiplex assay. **(G)** HERV-K10 expression was measured in PBMCs of chronically HBV-infected patients and healthy control PBMCs. For statistical analysis two-way-ANOVA was used (*p ≤ 0.05; **p ≤ 0.01; ***p ≤ 0.001; ****p ≤ 0.0001).

As previously noted, HERV-K10 expression is associated with various pathogenic conditions, and a MAG1-specific immune response has been implicated as a potential causative factor in several autoimmune diseases (9–11). Due to overlapping peptide sequences, a MAG1-specific immune response could also trigger molecular mimicry, potentially targeting host proteins such as IgG1-Fc (37). Although murine ERVs do not encode MAG1, its peptide regions share significant overlap with both murine and human IgG-Fc (**Figure 3A**). To investigate whether mice can mount a MAG1-specific immune response, we vaccinated them with an HAdV-C5-MAG1 vaccine (HAdV-C5-MAG1) (**Figure 3B**). One week after the HAdV-C5-MAG1 booster vaccination, mice were sacrificed, and the frequencies of MAG1-reactive CD4 and CD8 T cells, along with their antiviral effector functions, were assessed. Splenic lymphocytes were isolated, stimulated ex vivo with a MAG1-specific peptide, and analyzed by flow cytometry. In splenic lymphocytes, we observed an increase in MAG1-reactive IFNγ+ CD4 T cells compared to control mice (**Figure 3C**, **Supplementary Figure S3A**). However, MAG1-reactive IFNγ+ CD8 T cells remained unaffected (**Figure 3C**, **Supplementary Figure S3B**). Additionally, high levels of MAG1-specific antibodies were detected in the serum of MAG1 vaccinated mice compared to unvaccinated controls (**Figure 3D**). To assess whether a MAG1-specific CD8 T cell response could be observed later post-vaccination, we isolated and analyzed splenic and hepatic lymphocytes two weeks after the second HAdV-C5-MAG1 booster vaccination (**Figure 4A**). Again, the frequency of MAG1-reactive CD4 and CD8 T cells and their antiviral effector functions were evaluated through ex vivo stimulation with a MAG1-specific peptide, followed by flow cytometric analysis. In both splenic and hepatic lymphocytes from MAG1-vaccinated mice, we observed an increase in MAG1-reactive IFNγ+ CD8 T cells as well as TNFα+ CD8 T cells, compared to both control HAdV-C5-vaccinated and naive control mice (**Figure 4B**, **Supplementary Figure S4A**). Importantly, we observed a similar increase in HAdV-C5-reactive IFNγ+ and TNFα+ CD8 T cells in both splenic and hepatic lymphocytes of MAG1 and control HAdV-C5-vaccinated mice relative to naive controls (**Figure 4B**, **Supplementary Figure S4B**), suggesting that the vaccination titers were comparable across the groups. Furthermore, serum from MAG1 double-vaccinated mice exhibited high levels of MAG1-specific antibodies compared to HAdV-C5-vaccinated or naive control mice (**Figure 4D**), while HAdV-C5-specific serum antibodies were similar between both groups (**Figure 4D**).

To further test whether HBV-infected patients also possess an immune response against MAG1, we stimulated PBMCs from 5 chronically HBV-infected patients (chrHBV) or healthy individuals (hCo) with or without MAG1 peptide. Interestingly, MAG1 stimulation of PBMC of chrHBV patients resulted in an increased secretion of interleukin-6 (IL-6) and interleukin-1β (IL-1β) compared to mock-stimulated PBMC (**Figure 4E**). Healthy controls did not show differences in cytokine expression after MAG1 peptide stimulation (**Figure 4F**). These results also suggest a specific immune response to the MAG1 peptide derived from HERV-K10 gag1 in chronic HBV infection (**Figures 4E, F**). To further support the relevance of this response, we also analyzed HERV-K10 mRNA expression in PBMCs from the same cohort. Although the increase in HERV-K10 expression in chrHBV patients did not reach statistical significance, likely due to limited sample size, we observed a clear trend toward upregulation compared to healthy controls (**Figure 4G**). In conclusion, our results show that vaccination with HAdV-C5-MAG1 induced a MAG1-specific immune response in a mouse model, with an increase in MAG1-reactive CD4 and CD8 T cells and higher levels of MAG1-specific antibodies. In addition, chronic HBV patients showed elevated HERV-K10 expression and an immune response to MAG1 with elevated cytokine levels, suggesting a specific immune response in chronic HBV infection.

## Discussion

The tight regulation of HERVs plays a critical role during various stages of development. Emerging evidence also points to their involvement in the pathogenesis of diseases such as schizophrenia, dementia, cancer, and autoimmune disorders when these elements get reactivated (6). Several studies using exogenous viruses, including human immunodeficiency virus (HIV), influenza virus, hepatitis C virus (HCV) and adenoviruses, have shown that viral infections can trigger the expression of various HERV groups (14–17, 38). There is increasing evidence that HERV reactivation is involved in the innate immune response (39, 40). As HERVs are remnants of exogenous viruses, they can induce immune responses similar to those induced by natural infections. However, the mechanisms underlying the reactivation of HERVs in pathological conditions and how they may impact immunological pathways remain poorly understood.

In this study, we found that HERV-K10 activation was uniquely associated with hepatitis virus infection, as infection with SARS-CoV-2, TBEV, or another dsDNA virus, adenovirus 5 (Ad5V), did not induce its reactivation. This selective upregulation of HERV-K10 in response to certain DNA viruses may be attributed to the rapid replication of Ad5V, which could suppress HERV-K10 expression at an early stage. In our model, the full establishment of the hepatitis B virus (HBV) replication cycle takes 4 to 7 days, whereas Ad5V replication occurs much more rapidly, typically within hours, giving Ad5V the possibility to counteract HERV-K10 reactivation. Besides, virus recognition through specific Toll-like receptor (TLR) signaling pathways may contribute to differential reactivation of HERV-K10 in response to different DNA viruses. TLR2 is activated by HBV, HCV, and Ad5V, while TLR3, TLR4, and TLR7/8 are primarily activated by HBV and HCV (41–43). Activation of these receptors likely triggers common downstream effectors, such as TRAF3 and TBK1, which contribute to the upregulation of HERV-K10 and thus could make a difference in HERV reactivation.

Transcriptomic analysis of HBV-infected HepG2-NTCP-K7 cells revealed several HERV groups that are differentially expressed during HBV infection. Notably, we identified HERV groups, including LTR12C, MER41, and LTR5, which have previously been implicated in immune regulation. LTR12C has been shown to be regulated during infection with multiple viruses, including HIV-1 (38), and drives the expression of interferon-inducible genes such as Guanylate Binding Protein 2 (GBP2) and Guanylate Binding Protein 5 (GBP5), which encode broad-spectrum antiviral factors. MER41 has also been linked to the primate-specific interferon response (40, 44). In addition to these differentially expressed HERV groups, we identified several differentially expressed genes. REACTOME enrichment analysis revealed the involvement of these genes in immune-related pathways, and they were associated with various autoimmune diseases, including rheumatoid arthritis (45), systemic lupus erythematosus (46), and Aicardi-Goutieres syndrome (47), all of which have been linked to hepatitis infection.

Besides the reactivation of various HERV groups during hepatitis infection, our transcriptomic analysis identified the expression of two distinct HERV-K10 loci on chromosome 1, both containing the MAG1 domain. MAG1 is a HERV-K10 gag1-derived membrane-associated peptide and has been implicated in autoimmune diseases such as rheumatoid arthritis (RA) (11). A study found a significantly higher IgG antibody response to MAG1 peptide in RA patients compared to patients with other diseases or healthy controls, suggesting an antigen-driven immune response (11). This finding implies that MAG1 may be perceived as foreign by the immune system, potentially triggering immune cell activation and inflammatory responses. However, the study did not confirm that MAG1 itself drives the immune response. Our study is the first to experimentally demonstrate that MAG1 can induce both CD4+ and CD8+ T cell responses when presented to mice. Additionally, we observed the generation of MAG1-specific antibodies in our mouse model, which share overlapping peptide sequences with physiological proteins. Furthermore, MAG1 stimulation in chronic HBV-infected patients elicited a MAG1-specific immune response, as evidenced by the secretion of the pro-inflammatory cytokines IL-1β and IL-6. Both IL-1β and IL-6 are synergistic acute-phase cytokines that initiate systemic responses to infection and are associated with chronic inflammatory diseases (48, 49). Typically, IL-1β secretion requires two pathogen/damage-associated molecular pattern (PAMP/DAMP) signals. After the first signal, pro-IL-1β accumulates in inflammasomes, where it is cleaved by caspase-1 following the second signal (50, 51). Although our study demonstrates that MAG1 induces immune activation, the specific immune receptors or signaling pathways involved have yet to be identified. Future studies employing receptor binding assays, targeted MAG1 expression systems and *in situ* detection approaches are essential for elucidating the precise mechanisms underlying MAG1-mediated immunomodulation. Our findings suggest that chronic HBV infection leads to inflammasome accumulation, which is subsequently released upon HERV-K10 expression. This process likely recruits macrophages and monocytes to HBV-infected sites, exacerbating hepatocyte damage and viral replication. Moreover, elevated IL-6 levels have been linked to increased mortality in HBV patients (52) and contribute to chronic inflammation by reducing regulatory T cell (Treg) differentiation and increasing acute-phase proteins such as serum amyloid A (SAA). This exacerbates tissue damage and may contribute to diseases-like hepatocellular carcinoma (53–55). IL-6 is also implicated in several autoimmune diseases, and anti-IL-6 inhibitors, such as tocilizumab, are currently used to treat RA and are being tested in other autoimmune diseases (49, 52). The MAG1-induced increase in IL-6 represents an additional risk factor for autoimmune diseases associated with molecular mimicry.

The immunological roles of endogenous retroviruses are increasingly being recognized in the context of autoimmune diseases and various cancers (6, 40, 56–58). Notably, HERV expression has been found to influence inflammatory responses and immune surveillance as already described in the sections before. These genetic elements are involved in autoimmune diseases through various mechanisms, including molecular mimicry, whereby HERV-encoded proteins share epitopes with self-antigens, such as HERV-K10 MAG1 with IgG1 or HERV-W env with myelin, triggering cross-reactive responses (59), and the immune dysregulation caused by aberrant HERV expression, which disrupts T-cell tolerance and promotes autoantibody production, as seen in rheumatoid arthritis and lupus (60). However, HERVs also exhibit functions in oncogenesis, such as promoting tumor growth [e.g. HERV-K encodes the oncoproteins NP9 and REC (61)], and correlating with advanced tumor stage (62). Our study identified the activation of HERV-K10, a HERV-K subgroup, by HBV/HCV infection. Furthermore, we identified an immunomodulatory role for HERV-K10 MAG1 in the context of chronic HBV and HCV infection. As both infections are major risk factors for hepatocellular carcinoma (HCC), which is characterized by chronic inflammation and immune dysregulation (63), our findings suggest that virus-induced HERV activation could modulate antiviral immunity and contribute to long-term immune remodeling relevant for liver tumor development. While this is speculative at this stage, it warrants further investigation into the role of HERV-K10-derived antigens in the immunobiology of HBV/HCV-related HCC.

In conclusion, our study reveals the reactivation of multiple HERV groups following hepatitis infection, several of which are linked to immune reactivation and autoimmune diseases. Notably, we identified the upregulation of HERV-K10, which encodes MAG1, a peptide previously associated with rheumatoid arthritis. MAG1 expression in mice elicited a T-cell response and the production of MAG1-specific antibodies. Furthermore, chronic HBV infection in patients was associated with a MAG1-specific immune response. Together, these findings suggest that a MAG1-driven immune response during hepatitis infection may contribute to the pathogenesis of autoimmune diseases associated with hepatitis. Although we demonstrated robust immune activation induced by MAG1, our current study does not directly address whether this activation is protective or pathogenic in the context of liver disease associated with HBV or HCV. Clarifying this dual potential is an important area for future research, which could help to define the functional role of HERV-K10 in viral hepatitis and liver pathology more clearly. Further investigation of the mechanisms leading to HERV-K10 expression could pave the way for novel therapeutic strategies for chronic HBV, potentially reducing inflammation and the risk of secondary complications such as autoimmune diseases in affected patients.

## Limitations

Additional experiments involving RNA viruses, such as TBEV and SARS-CoV-2, suggest that the upregulation of HERV-K10 is not an inherent consequence of innate immune activation, but rather a characteristic of HBV and HCV infections. However, we cannot rule out the possibility of immune signaling contributing to the process, and future studies using HBV mutants or immune-modulatory models will be necessary to distinguish between replication-dependent and immune-mediated mechanisms. Although our patient cohort was small and detailed clinical metadata was unavailable for all individuals, we nevertheless observed consistent trends and statistically significant differences, which support the robustness of our findings. Future studies involving larger cohorts of patients with comprehensive clinical data will be essential to validate and expand upon these observations, including potential correlations with disease stage or immune status.

## Data Availability

The original contributions presented in the study are included in the article/**Supplementary Materials**, further inquiries can be directed to the corresponding author/s. The RNA-seq data presented in this paper can be accessed under the GEO accession number GSE294437.

## Supplementary material

Supplementary Figure 1 HERV-K10 expression upon Ad5V infection and in primary HBV infected Hepatocytes **(A)** Relative HERV-K10 mRNA expression levels of HBV infected primary hepatocytes (PHH). **(B)** Relative pgRNA expression of HBV-infected PHH. **(C)** Relative HERV-K10 mRNA expression of Ad5V infected HepG2-NTCP-K7 compared to non-infected control. **(D)** Depicted is luminescence in relative units (RLU) from Ad5V infected HepG2-NTCP-K7 compared to non-infected control. **(E)** The successful infection of cells by SARS-CoV-2 or TBEV at an MOI of 0.1 was measured via qRT-PCR. **(F)** The relative expression of HERV-K10 mRNA was measured using qRT-PCR in cells infected with SARS-CoV-2 or TBEV, respectively, compared to uninfected cells. For statistical analysis two-tailed unpaired T-test was used (*p ≤ 0.05, **p ≤ 0.01; ***p ≤ 0.001; ****p ≤ 0.0001).

Supplementary Figure 2 Genes reactivated upon HBV infection are associated with autoimmune diseases. **(A)** Volcano blot of differentially expressed (DE) genes upon HBV infection. The significance level (dotted horizontal line) is 0.05. **(B)** Network interactions between genes and autoimmune diseases. Depiction of a network of interactions between the identified gene set and autoimmune diseases was generated by the multifactorial CIDeR database.

Supplementary Figure 3 Immune response upon HERV-K10 derived MAG1-vaccination in mice. The Figure shows the individual flow cytometry plots corresponding to **Figure 3**. **(A)** MAG1 reactive CD4 T cells and **(B)** MAG1 reactive CD8 T cells.

Supplementary Figure 4 Immune response after HERV-K10 derived MAG1-vaccination in mice. The Figure shows the individual flow cytometry plots corresponding to **Figure 4**. **(A)** MAG1 reactive CD8 T cells and **(B)** AD5V reactive CD8 T cells.
