# Supplementary figures and images for: HERV-K10 as a mediator of immune modulation in hepatitis infections
Source: Front Immunol. 2025 Sep 17;16:1624774. doi: 10.3389/fimmu.2025.1624774 (PMC12484233; doi:10.3389/fimmu.2025.1624774)

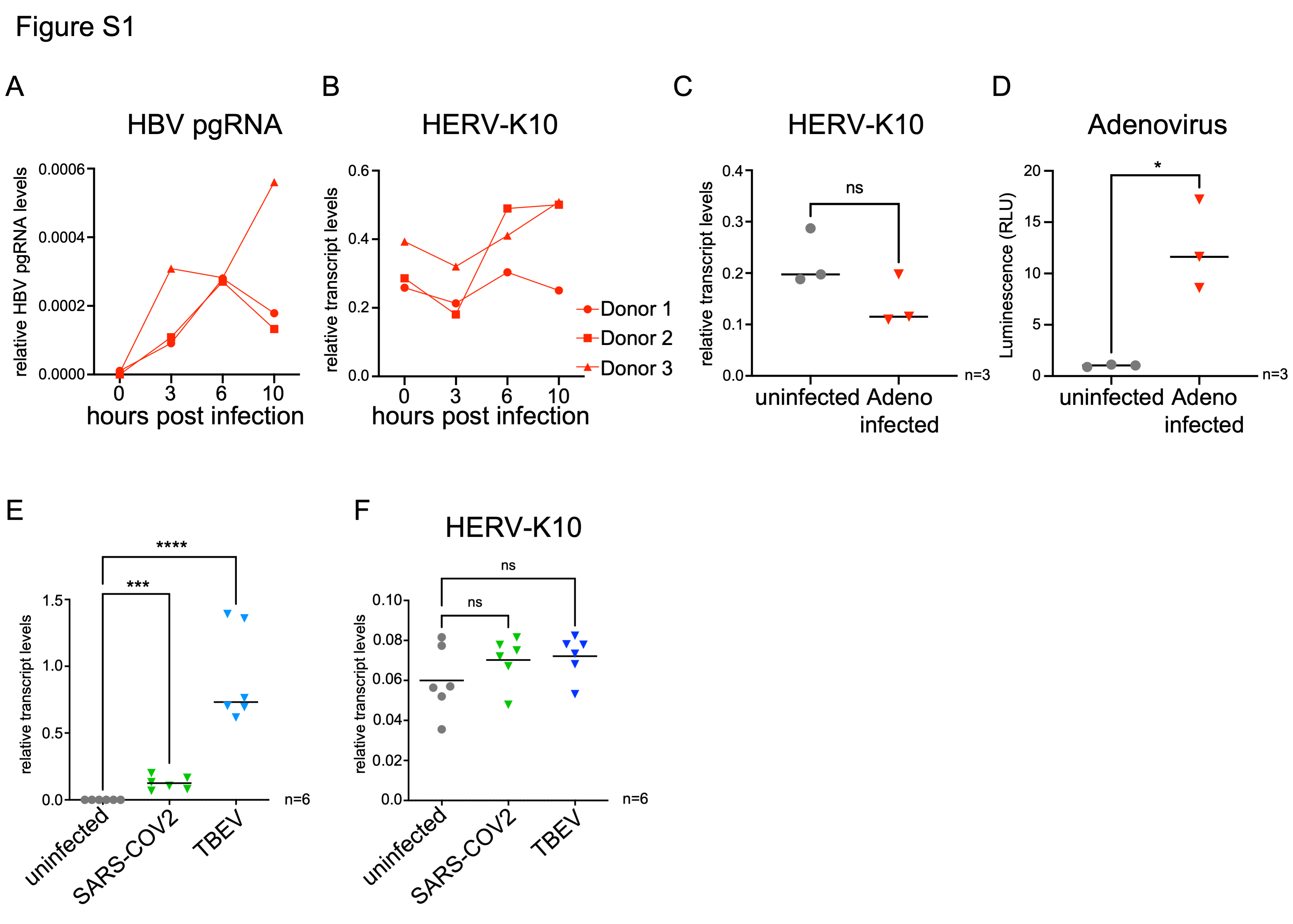

Supplement: Supplementary Figure 1 — HERV-K10 expression upon Ad5V infection and in primary HBV infected Hepatocytes (A) Relative HERV-K10 mRNA expression levels of HBV infected primary hepatocytes (PHH). (B) Relative pgRNA expression of HBV-infected PHH. (C) Relative HERV-K10 mRNA expression of Ad5V infected HepG2-NTCP-K7 compared to non-infected control. (D) Depicted is luminescence in relative units (RLU) from Ad5V infected HepG2-NTCP-K7 compared to non-infected control. (E) The successful infection of cells by SARS-CoV-2 or TBEV at an MOI of 0.1 was measured via qRT-PCR. (F) The relative expression of HERV-K10 mRNA was measured using qRT-PCR in cells infected with SARS-CoV-2 or TBEV, respectively, compared to uninfected cells. For statistical analysis two-tailed unpaired T-test was used (*p ≤ 0.05, **p ≤ 0.01; ***p ≤ 0.001; ****p ≤ 0.0001). [file Image1.jpeg]

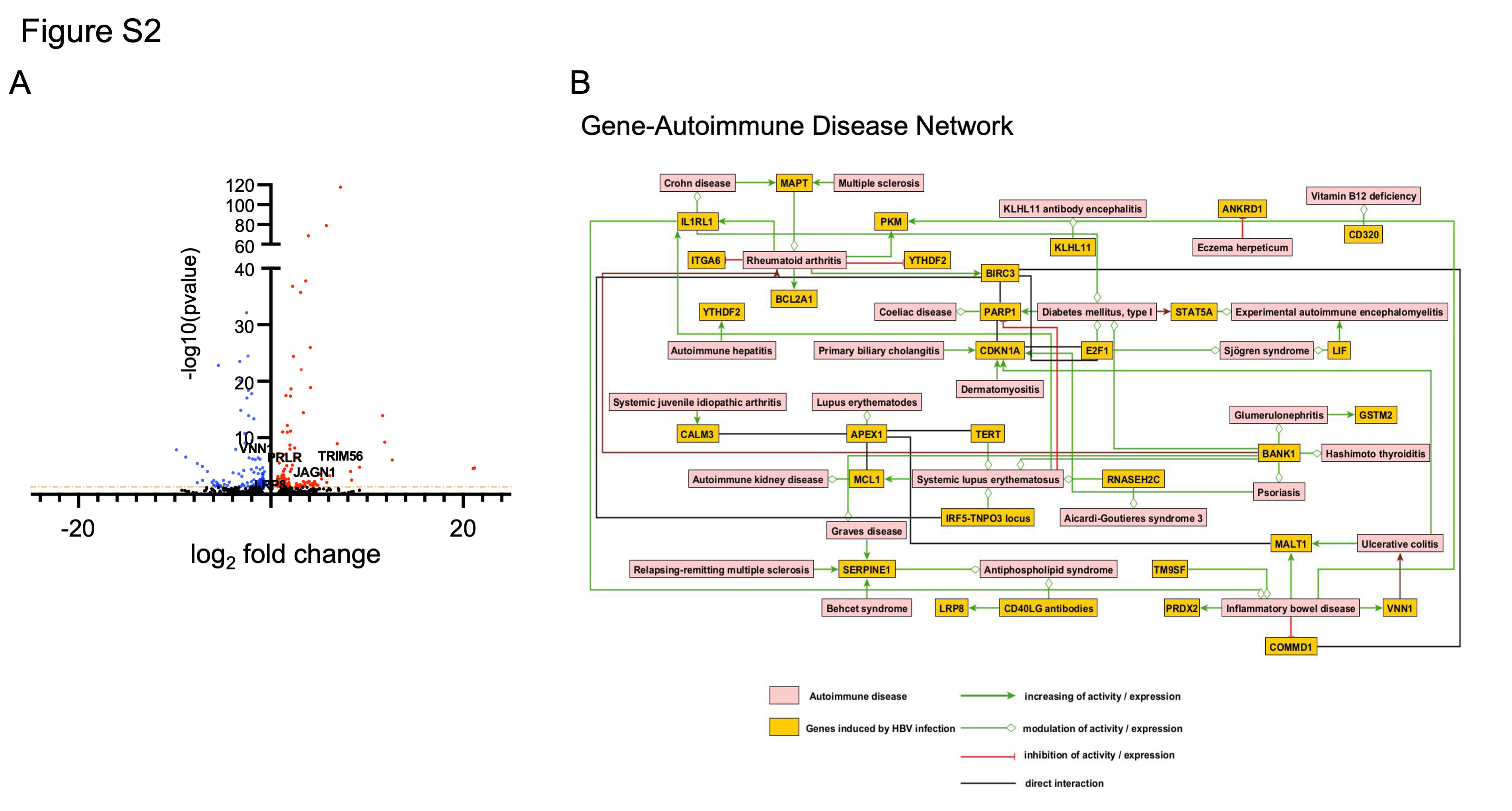

Supplement: Supplementary Figure 2 — Genes reactivated upon HBV infection are associated with autoimmune diseases. (A) Volcano blot of differentially expressed (DE) genes upon HBV infection. The significance level (dotted horizontal line) is 0.05. (B) Network interactions between genes and autoimmune diseases. Depiction of a network of interactions between the identified gene set and autoimmune diseases was generated by the multifactorial CIDeR database. [file Image2.jpeg]

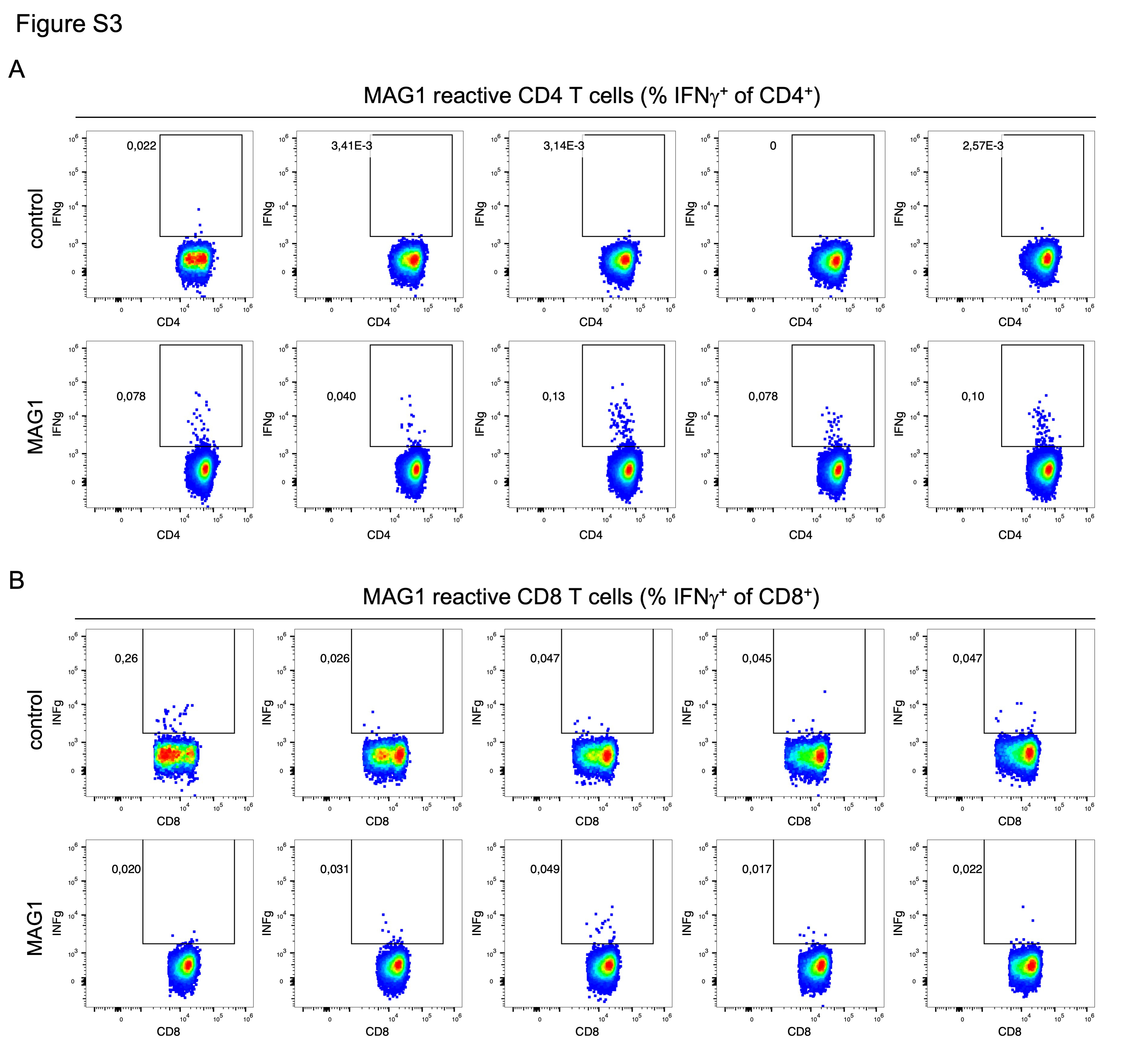

Supplement: Supplementary Figure 3 — Immune response upon HERV-K10 derived MAG1-vaccination in mice. The Figure shows the individual flow cytometry plots corresponding to Figure 3 . (A) MAG1 reactive CD4 T cells and (B) MAG1 reactive CD8 T cells. [file Image3.jpeg]

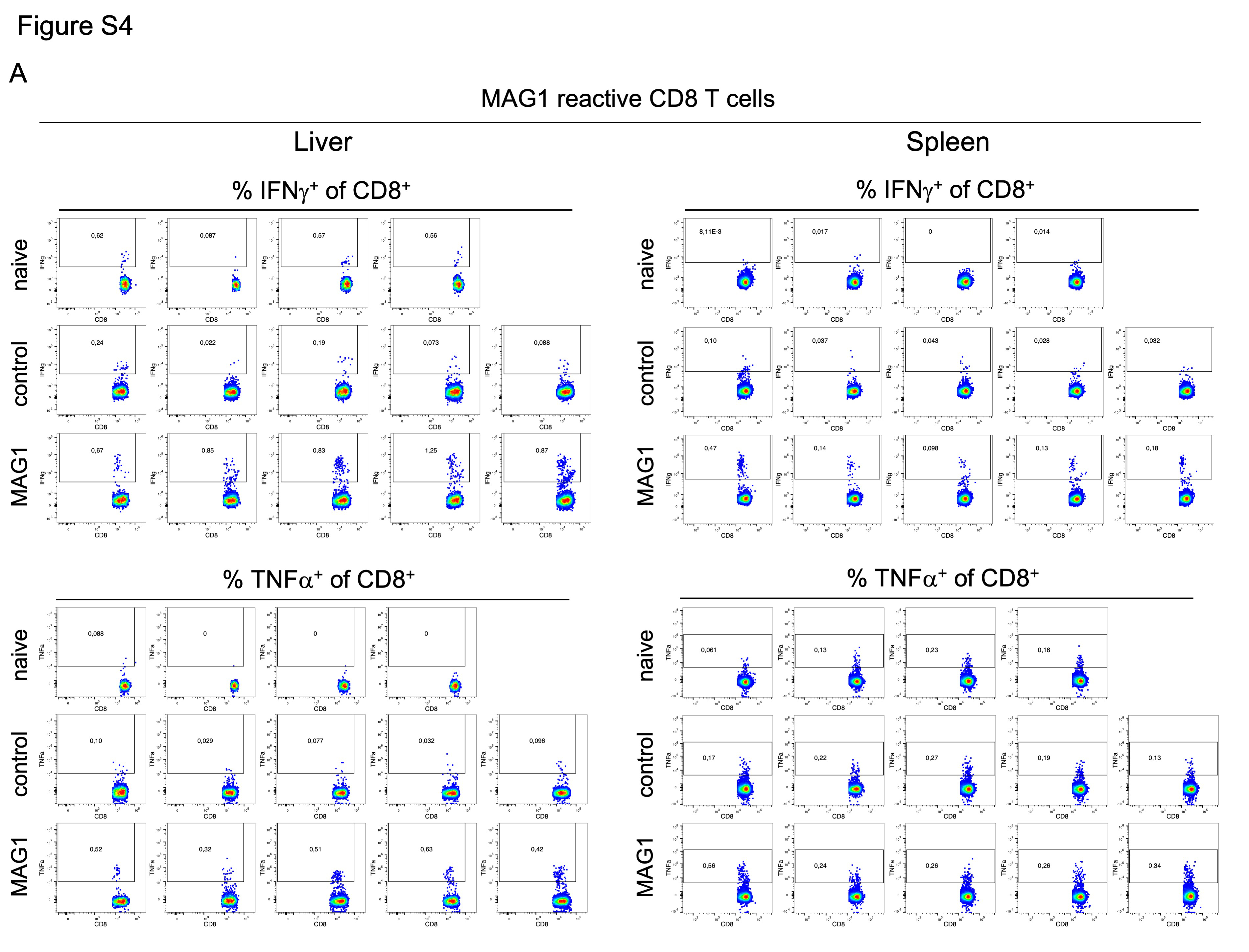

Supplement: Supplementary Figure 4 — Immune response after HERV-K10 derived MAG1-vaccination in mice. The Figure shows the individual flow cytometry plots corresponding to Figure 4 . (A) MAG1 reactive CD8 T cells and (B) AD5V reactive CD8 T cells. [file Image4.jpeg]

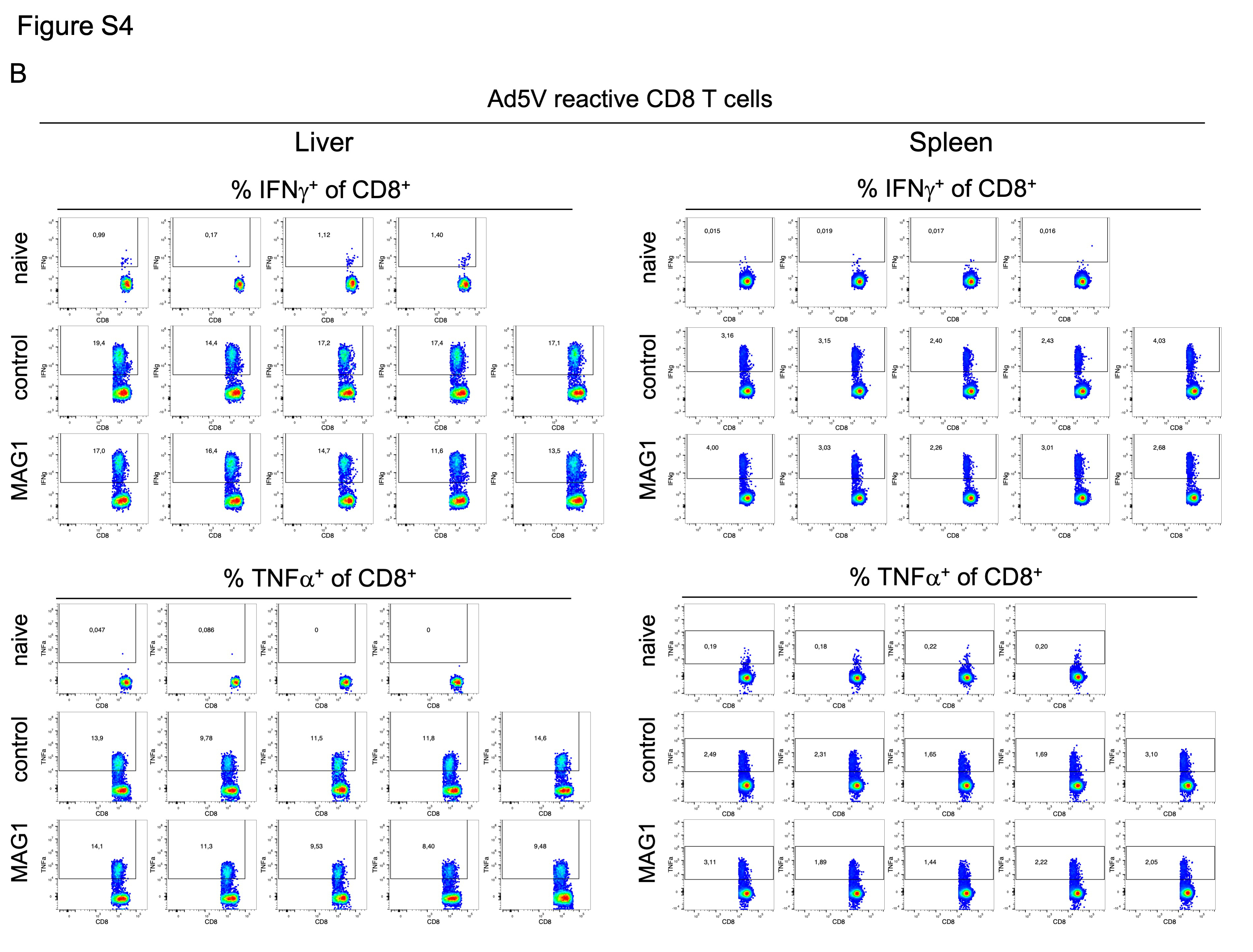

Supplement: Supplementary file 5 [file Image5.jpeg]
